# Animal-based food systems are unsafe: severe acute respiratory syndrome coronavirus 2 (SARS-CoV-2) fosters the debate on meat consumption

**DOI:** 10.1017/S1368980020002657

**Published:** 2020-12

**Authors:** Michelle Cristine Medeiros Jacob, Ivanilda Soares Feitosa, Ulysses Paulino Albuquerque

**Affiliations:** 1 Laboratório Horta Comunitária Nutrir, Nutrition Department, Universidade Federal do Rio Grande do Norte, Campus Universitário, Natal, Rio Grande do Norte 59.078970, Brazil; 2 Laboratório de Ecologia e Evolução de Sistemas Socioecológicos, Botany Department, Universidade Federal de Pernambuco, Recife, Pernambuco, Brazil

**Keywords:** Food and nutrition security, Food safety, Food systems, Meat

## Abstract

**Objective:**

The current pandemic restarts a debate on permanently banning wildlife consumption in an effort to prevent further public health threats. In this commentary, we offer two ideas to enhance the discussion on foodborne zoonotic diseases in food systems.

**Design:**

First, we focus on the probable consequences that the loss of access to wildlife could cause to the status of food and nutrition security of many people in developing countries that rely on bushmeat to subsist. Second, we argue that all animal-based food systems, especially the ones based on intensive husbandry, present food safety threats.

**Conclusion:**

To ban the access to bushmeat without a rational analysis of all human meat production and consumption in the global animal-based food system will not help us to prevent future outbreaks.

The proximal origin of severe acute respiratory syndrome coronavirus 2 (SARS-CoV-2) involves bushmeat, such as bats (*Rhinolophus affinis*) and Malayan pangolins (*Manis javanica*)^(1)^, but we have a lack of evidence about the intermediary sources of origin and transfer to humans^(2)^. Bushmeat or wild meat is defined as meat derived from any wild animal, especially non-aquatic vertebrates, harvested for subsistence or trade, most often illegally^(3)^. It is not the first time that a virus that originated from animals infected humans. Up to 73 % of emerging infectious diseases in humans are of zoonotic origin, most of which originate in wildlife^(4)^. In the last few years, attention has been paid to epidemic zoonoses such as avian influenza (H5N1) and the SARS.

SARS-CoV-2 fosters a debate on permanently banning wildlife consumption in an effort to prevent further public health threats related to foodborne zoonotic diseases^(5)^. Such zoonoses are a global menace, and little is known about the mechanisms that make these diseases emerge. Spatial modelling demonstrates the significant risk of these zoonoses appears in regions of tropical forests, which are also associated with a high diversity of mammals^(6)^. In these regions, there is great cultural diversity associated with these animals and many thousands of people whose ways of life depend on them.

We offer two ideas to contribute to this discussion. First, we argue that the loss of access to wildlife can compromise the status of food and nutrition security of many people in developing countries that rely on bushmeat to subsist. Second, we argue that environmental impacts bred by factory farming are important drivers of outbreaks of foodborne zoonotic diseases in animal-based food systems. Our main arguments and conclusions are summarised in Fig. 1. For better comprehension, we start each argument by presenting the concepts of food and nutrition security (FNS) and food safety.

**Fig. 1:**
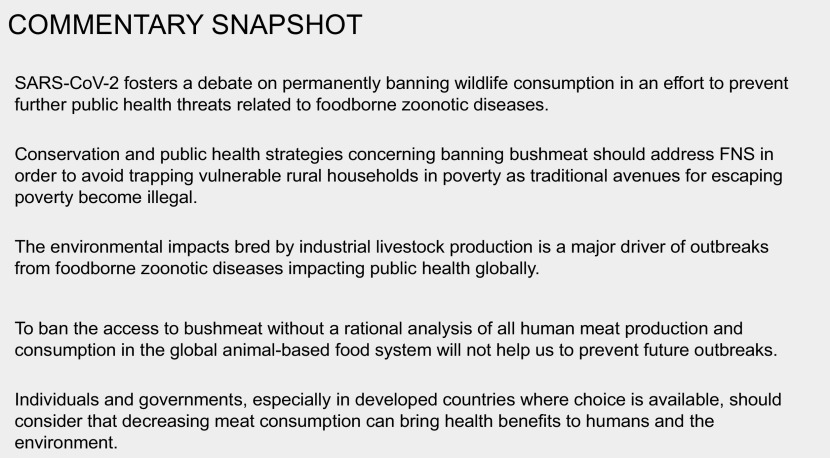
To ban bushmeat consumption is not the solution to prevent future outbreaks in animal-based food systems. FNS, food and nutrition security; SARS, severe acute respiratory syndrome

## Bushmeat contributes to food and nutrition security of people in developing countries

FNS is defined as a state in which people access proper food, in quantity and quality, without compromising other basic needs, while safeguarding cultural diversity, and economic, social and environmental sustainability^(7)^. This concept is sustained by four critical pillars: availability (reliable food supply), accessibility (resources to obtain proper food), utilisation (food quality and health conditions) and stability (permanent access to food). In our current global food systems, whose high productivity is based on models with controversial socio-environmental and human health impacts, we still need to deal with endemic hunger due to political, economic and social constraints^(8)^. One of the most unfortunate human achievements is that one-third of all food produced globally is lost or goes to waste, while a little over 820 million people suffer from hunger. Most of this vulnerable population lives in Africa and Latin America, where the rates of undernourishment have increased in recent years^(9)^. By analysing the current state of evidence, we argue that bushmeat contributes to the FNS of millions of these people by three central channels: by improving diets, by generating income and by protecting cultural heritage.

First, bushmeat makes a significant contribution to diets because, in many situations of severe food insecurity, it is one of the only foods accessible to people, besides being the primary source of protein. Bushmeat contributes up to 90 % of the animal protein consumed in certain rural regions of West and Central Africa and over 20 % of that eaten by several indigenous groups in the Amazon^(3)^. Bat bushmeat is a core dietary component for people living in Madagascar^(10)^. In several South American countries, the meat of capybara (*Hydrochoerus hydrochaeris*) is an essential resource for the subsistence of many traditional communities^(11)^. In Ghana, fruit bat bushmeat provides an at-cost protein source when agricultural production drops in the dry season^(12)^. In São Tomé, the rural population is largely dependent on the protein of wild animal species^(13)^. Finally, in several countries in the Congo Basin, the dependence on bushmeat protein is emphasised by the fact that the countries do not produce enough amounts of non-bushmeat protein to feed their populations^(14)^. To date, from a nutritional point of view, there is no consistent data to compare bushmeat and domesticated meat composition values, considering species and consumed parts.

Besides, bioavailability makes animal sources of foods higher in macro and micronutrients than vegetable sources, which in the case of populations with limited access to food needs to be considered. Some studies assess the impact of bushmeat in the nutritional status of humans. Golden *et al.* ^(15)^, for example, in a prospective longitudinal cohort of seventy-seven preadolescent children in rural northeastern Madagascar, show that consuming more animal wildlife was associated with significantly higher Hb concentrations. Removing access to wildlife would induce a 29 % increase in the number of children suffering from anaemia and a tripling of anaemia cases among children in the poorest households. Other studies indicate that wild natural resources may be central to FNS coping strategies of orphans and vulnerable children affected by HIV/AIDS. Considering the heightened nutritional needs of children, as well as poverty and disease, McGarry and Shackleton^(16)^ show that bushmeat represents the last freely attainable protein food source available to them.

Second, bushmeat represents a full-time source of income in many households. Even in cases in which bushmeat is not the primary source of income, it can serve as a safety net that complements household income in challenging times (e.g. droughts, unemployment and illness) or a source of extra cash for special needs (e.g. school fees, festivals and funerals)^(17)^. On the other hand, bushmeat trade can also figure as a profitable global industry that threatens certain animal species with extinction. Conservation strategies, for this reason, should consider the complexity of its uses. Uses of bushmeat vary from subsistence-based rural consumption and subsistence-commercial hunting to a luxury commodity in urban areas^(18)^. In a study about economic and wildlife uses conducted in 2000 households from ninety-six settlements in Ghana, Cameroon, Tanzania and Madagascar, Golden *et al.* ^(15)^ argue that wildlife hunting and consumption in rural areas increase when alternative livelihoods collapse. They also found an opposite pattern in the urban context, where there was a consistent relationship between wealth and wildlife consumption. For these reasons, efforts to conserve wildlife will need to study rural and urban consumers and the drivers and patterns of consumption and trade.

Third, bushmeat hunting and consumption are embedded in several communities as their cultural heritage^(3)^. And FNS is also about cultural acceptability, as shown in Comment 12^(19)^ on *The Right to Adequate Food* by the United Nations. It says: FNS *implies the need also to consider, as far as possible, perceived non-nutrient-based values attached to food and food consumption and informed consumer concerns regarding the nature of accessible food supplies* (p. 3). The flavour perception, for example, has a strong cultural background that can play a significant role in the acceptance of food by human groups. In a study about hunters’ preferences and perceptions as drivers to hunt in a semiarid ecosystem, Chaves *et al*.^(20)^ found that flavour preference can increase the odds of a species being hunted almost 100 %. Furthermore, in several traditions around the world, bushmeat is a central part of rituals, traditional ceremonies, linked with social status and power, and ancestral ties. Frequently, bushmeat is considered sacred, and so its consumption is taboo and forbidden. Some studies defend that social taboos, as informal institutions that determine human behaviour, can be useful in partnership designs to prevent bushmeat consumption^(21)^. On the contrary, Ndlovu^(22)^ warns from his experience in Zimbabwe that reinforcing taboos to discourage the eating of some meats can lead to people suffering from starvation. The effects of cultural acceptability on FNS and biological conservation deserve better comprehension to improve political decision-making in this field.

In terms of conservation, several considerations need to be made, as we need to clearly discern whether FNS is, in fact, dependent on bushmeat to improve diet. Brazil can be a typical case in this sense because while in the Amazonian forest, there seems to be a strong dependence on bushmeat, in the semiarid portion, consumption appears to be related only to cultural heritage without direct implications for the FNS of the people who live there^(23,24)^.

## All animal-based food systems offer food safety threats

**Fig. 2:**
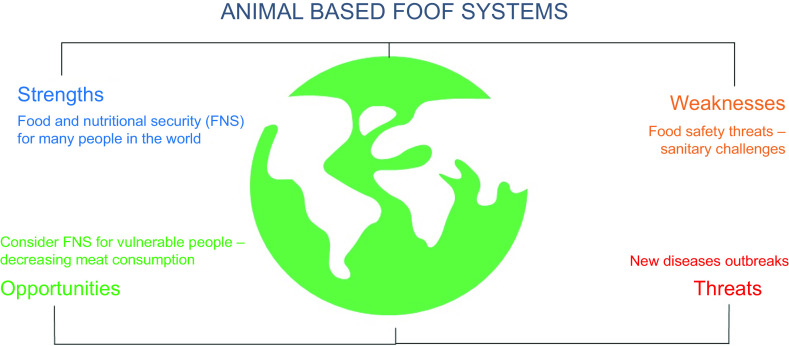
A summary of animal-based food systems strengths, opportunities, weaknesses and threats

Food safety refers to conditions and practices that preserve the quality of food to prevent contamination and foodborne illnesses across the whole food system.^(25)^ SARS-CoV-2 brought the topic of consumption of bushmeat and zoonotic diseases back to the agenda since three-fourths of emerging infectious diseases to humans have a zoonotic origin (includes Ebola virus, hantaviruses, SARS-coronaviruses, HIV-1, HIV-2 and influenza)^(26)^. However, the proposition to ban bushmeat as a measure of food safety is not as simple as it seems to be. This argument is narrow for two main reasons. First, it does not consider that rural and urban communities living in poverty rely on bushmeat to subsist. Second, it is constructed upon ethnocentric bias that undermines our ability to analyse the whole picture: Our westernised intensive industrial food system is a significant part of the problem. *Hell is other people*, to paraphrase the idea of the philosopher Jean-Paul Sartre. No, it is not just the others, we are hell, also. In Fig. 2, we show a summary of animal-based food systems strengths, opportunities, weaknesses and threats.

Pathogens that we see as new, previously existed in nature. For example, HIV-1 and 2 have chimpanzees and sooty mangabey as reservoir species in the wild, as does anthrax in species of ungulates^(26)^. Its spillover success to receptive hosts depends on environments that offer favourable conditions^(27)^. The analysis of epidemiological characteristics of viral transmission between animals and people shows that a series of high-risk human activities have allowed viruses to spread in the past, enabling the approximation between wild species, domesticated animals and people^(28)^. Industrial livestock production is one of these activities. Much of the deforestation of tropical forests is justified by promoting areas for raising livestock^(29)^. According to Volpato *et al.* ^(30)^, the invasion of human activities in forests is among the main factors that offer the necessary conditions for the transmission of viruses to humans, through the proximity of bats and intermediate hosts, including cattle. After the SARS epidemic in 2002, there was a growing interest in investigating coronavirus in bats. It is estimated that more than 200 new types have been identified, and 35 % have already been sequenced. This discovery makes bats the main reservoirs and transmitters of coronavirus, although many other animals can also perform this function^(31)^. A notable example was observed in eastern Australia, where bats after the forest were cleared for pasture, remained perched in the trees cut down. This proximity to domesticated animals allows them to transmit coronavirus through their urine and faeces^(30)^.

**Fig. 3:**
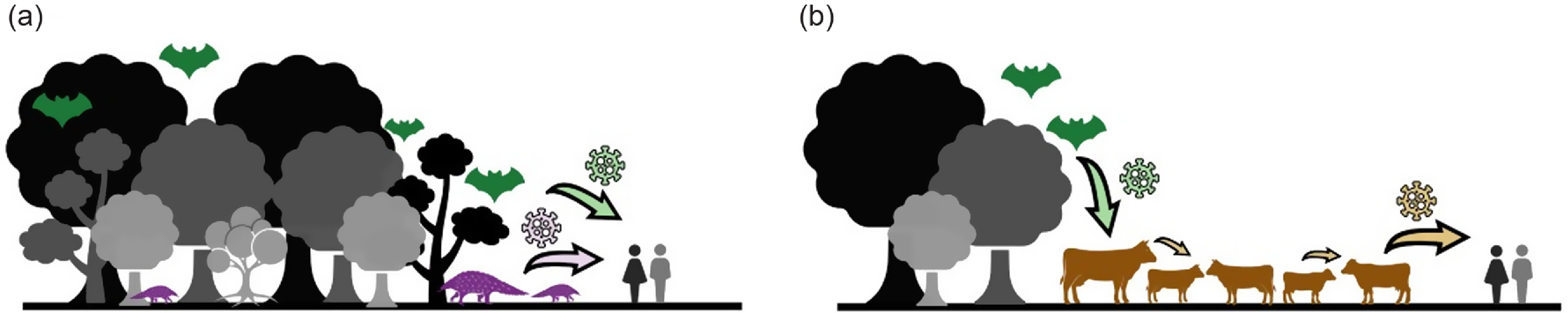
The path of the virus from the wild ecosystems to human beings. (a) Wildlife consumption; (b) intensive husbandry

The invasion of humans in settlements and the establishment of agriculture in natural ecosystems result in the expansion of ecotones, that is, transition zones between adjacent ecological areas where species are artificially mixed^(32)^. As human occupy and transform these environments, they disrupt the ecology of wild animals, altering the ecosystem balance and increase the likelihood that viruses (see Fig. 3) will find intermediate hosts (wild or domesticated)^(30)^. For example, there is evidence of a relationship between the increased spread of the encephalitis virus and production of rice and pig culture in Southeast Asia^(4)^. Deforestation, habitat destruction and mining are drivers of the resurgence of rabies spread by the hematophagous bat to humans in the Amazon^(31)^. The emergence of haemorrhagic fever in central-west Argentina in the 1950s was linked to the development of agricultural activities, mainly the cultivation of maize that sustains its main reservoir (*Calomys musculinus*)^(33)^. The expansion of agricultural frontiers promotes the degradation of habitats, bringing humans and cattle closer to vectors and sylvatic cycles of possible zoonotic pathogens.

Repeated outbreaks associated with the production of meat, eggs and milk demonstrate that the risks of pathogens arising from livestock activity are maximised by intensive production. Additionally, the human contact with hunting and preparing wild animals for consumption has led to a wide transmission of diseases. The volume of consumption of wild meat for the subsistence of humans is less than that of domestic origin. The substitution of wild meat with meat produced from domesticated animals must be considered carefully because it can favour the emergence of new pathogens^(34)^. Many foodborne zoonoses are enzootic in cattle (e.g. bovine tuberculosis). Livestock can become an intermediate or amplifier where pathogens can evolve and overflow to humans^(4)^. Factors such as stocking rates, mixtures of species and lack of disease control methods promote the spread of this type of disease and new infections in humans^(34)^.

Animals can be a source of pathogens in food products through faecal contamination, water and zoonotic diseases transmitted directly from animals to humans, among others. The conditions in which thousands of animals live on industrial farms are unhygienic. Allied to this, stress, lack of exercise and unnatural diets increase the chances of their immune system being compromised, leading to being affected by diseases^(35)^. The sum of all these factors turns breeding grounds into perfect places for the spread of new pathogens. To combat this problem, these animals are subjected to the use of antibiotics regularly, which makes them many times more resistant to certain types of pathogens^(35)^. Although these environments are reservoirs for zoonotic diseases, studies show that ranchers are aware that their management practices are responsible for significant environmental impacts and the consequent transmission of these diseases to several species of domesticated animals^(36,37)^.

Cases of zoonotic diseases that were also hosted by other domesticated animals were responsible for several epidemics in different parts of the world (e.g. H5N1 avian influenza, Nipah virus in pigs). Therefore, SARS-CoV-2, instead of being a scenario to feed our prejudice and cultural stigmas towards others, can be an opportunity to analyse with a critical and honest view our appetite for meat^(20)^.

## Conclusion

Conservation and public health strategies concerning banning bushmeat should address FNS to avoid trapping vulnerable rural households in poverty as traditional avenues for escaping poverty become illegal. Furthermore, SARS-CoV-2 can be a warning that indicates the need for a holistic and transformative shift in our food system. These changes should include the development of food production methods capable of limiting environmental degradation. Finally, considering that all animal-based food systems offer food safety threats, individuals and governments, especially in developed countries where choice is available, should consider that decreasing meat consumption can bring health benefits to humans and the environment.
